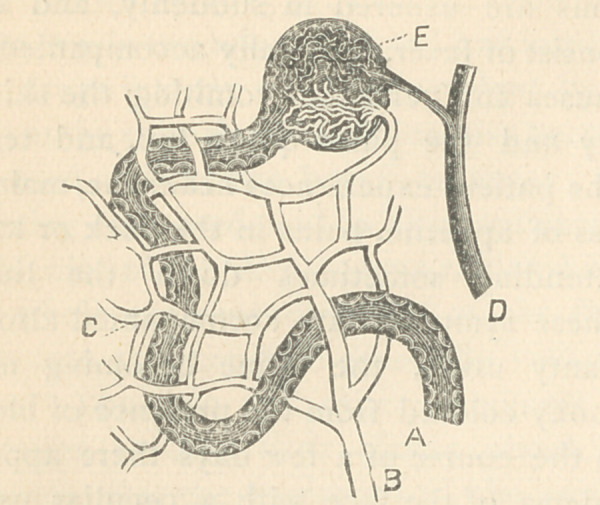# Nephritis Secondary to Aortic Regurgitation

**Published:** 1888-04

**Authors:** John A. Robison

**Affiliations:** 428 Washington Boulevard


### NEPHRITIS SECONDARY TO AORTIC REGURGITATION.

BY JOHN A. ROBISON, A. M., M. D.

A Clinical Lecture Delivered at Cook County Hospital, Reported by William Whitford, M. D.

GENTLEMEN: The patient to whom I direct your attention this afternoon was admitted to the hospital on the third day of January. I will simply state, without any preliminary remarks about the history of the patient, that the case is one of nephritis due to aortic regurgitation. Albuminuria is due to three general causes—the exposure of the patient to cold or inclement weather, or to some septic material which enters the circulation and causes an inflammation of the kidney, and, finally, it may be secondary to disease in other organs, as valvular heart-disease, or constitutional disease. The case under consideration belongs to the third class of causes, the cause being aortic regurgitation. The heart being unable to carry on perfectly the systemic circulation, there is derangement of the circulation in the kidneys, causing disease, as we will see later.

The symptoms which occur in nephritis vary according to its acuteness or chronicity. In acute Bright's disease the symptoms are ushered in suddenly, and they consist of fever, generally accompanied by nausea and persistent vomiting; the skin is dry and the pulse quick, full, and tense. The patient experiences headache, malaise, loss of appetite, pains in the back or loins, extending sometimes down the limbs. These symptoms are accompanied also by scanty urine, the urine becoming more smoky-colored from the presence of blood. In the course of a few days there appears œdema of the face, with a peculiar pallor or pasty complexion, and this œdema extends to other parts of the body, especially if the patient be anæmic or debilitated. The onset of chronic nephritis is gradual and insidious, the œdema of the face, with œdema of the extremities, first calling attention to the presence of the disease. In both the acute and chronic disease the urine becomes albuminous; the microscope may reveal the presence of tube-casts, blood corpuscles, fatty oil-globules, etc., according to the stage of the disease. I think there are not a few students who have not a clear idea as to the mode of production of albuminous or bloody urine, and I wish to try and explain this point to you.

I desire first to say a few words concerning the blood supply of the kidneys, in order to illustrate in as clear a manner as possible this subject.

The location of the kidneys is such that the renal artery enters toward the centre or hilum of each kidney. When this artery enters the kidney it subdivides into a number of smaller branches which approximate in number the medullary pyramids.

These branches enter the substance of the kidney between the papillæ, and when at the bases of the pyramids, at the junction of the medullary and cortical substances, they curve inward, forming incomplete arches, giving off two sets of branches, one set running between the malpighian bodies and giving off lateral twigs which form the *afferent* vessels to the uriniferous tubes.

If you will study the following figure, modified from Johnson's "Bright's Diseases," you will be able to better understand the course the blood takes. The dilated end of the uriniferous tube (A), or Bowman's capsule, is pierced by the *afferent* vessel (D), which brakes up into a tuft of capillaries, the glomerulus, which reunites into an *efferent* vessel and makes its exit from the capsule, and immediately breakes up into a plexus of capillaries (C) surrounding the convuluted tubes, and again uniting forms the vein (B) which empties into the interlobular veins. The tuft of capillaries in Bowman's capsule is covered by the same cells as line the capsule. It must be remembered that the efferent vessel is smaller than the afferent and breakes up into a plexus. This has the effect of slowing the blood current and increasing the blood pressure in the glomerulus.

Now you can readily see that whenever the blood current in the kidney falls below that which is necessary to nourish the cells covering the glomerulus, they will die, be exfoliated, and thus allow the albumen of the serum of the blood to filter into the tubules along with the saline and watery elements that form the urine. You can also realize how easily blood may escape from these tufts into the tubules and appear in the urine. The conditions which will cause death of these epithelial cells are, first, diminished arterial pressure causing anæmia of the kidneys; second, inflammation of the kidney, producing derangement of the circulation sufficient to cause degeneration of the epithelium of the glomerulus and tubules; third, various chemicals, drugs, and septic materials derange the renal circulation in like manner; fourth, in spasmodic diseases there may be such a disturbance of the renal circulation as to cause temporary anæmia of the kidney, and, finally, in venous congestion, as in valvular lesions, when there is obstruction to the systemic circulation, the blood current is wonderfully altered, because there is a lessened arterial pressure in this arterial twig (D), on account of the weakness of the heart, and secondly on account of the congestion of the veins (C) pressing back the blood and causing stagnation in the capillary tufts (E). If any of the foregoing causes operate, you see it causes the death of the epithelial linings of the tubules and the escape of albumen, and blood at times, and these will be found in the urine. I think it simplifies matters for us to remember that whatever the cause of nephritis, the real seat of the danger is in the capillary tuft in Bowman's capsule, although, of course, we must remember that all the tissues may undergo change. Degeneration of the epithelial structures is the great source of albuminuria.

In acute Bright's disease and chronic parenchymatous nephritis, the degeneration of the epithelia is primary; in cirrhotic Bright's disease, the epithelial degeneration is caused by the cellular infiltration of the inter-tubular connective tissue, and the partial or complete obliteration of the tubules, and death of the epithelial structures, by reason of this growth and contraction of connective tissue. In the amyloid kidney, the glomeruli, arterioles, tubules, and all the tissues become infiltrated with amyloid material.

I will simply say a few words about the general treatment for all forms of nephritis. The first indication, of course, would be complete rest both for the kidneys and the body, the patient being placed at rest in order to obviate as much as possible the necessity of the kidneys doing much work. In the next place, inasmuch as the skin is generally inactive, we should use diaphoretics to assist elimination. You may also in suitable cases stimulate elimination by means of cathartic remedies. In this way you prevent the necessity of eliminating poisonous materials from the blood via the kidneys. Diaphoresis may be induced by hot baths, vapor or Turkish baths. You should also give diluents, and the best diluent, of course, is the purest water. Mineral waters, especially in cases of acute albuminuria, are objectionable, because if the water contains very much mineral matter, as it sometimes does, it is irritating to the kidneys, and during all stages of the disease we desire to avoid irritants. If there be much pain your patient must be kept as easy and comfortable as possible with opium. You should also avoid the administration of diuretics, which are irritating to the kidneys, such as the balsamic preparations, which are very apt to produce hæmaturia. When the urine becomes nearly normal and is secreted more freely, you may give remedies specially directed to influence the kidneys locally. The remedies which have been given so far in these cases are more or less empirical. They are principally tincture of iron, which is used on account of its local astringent as well as diuretic action, and the tincture of iodine. Digitalis is a remedy of prime importance, because it raises the blood pressure in the kidneys and assists in restoring the nutrition of the epithelial cells, as well as being a potent diuretic.

In the case of this patient you must remember the fact that the nephritis is the result of aortic regurgitation; therefore we must try and remove the cause, by inducing compensatory hypertrophy of the heart. He has been taking for some time ten minims of the tincture of digitalis four times a day; tincture of iron, fifteen minims, four times a day. On admission, the amount of albumen present was 10 per cent. He has also been given during this time Rochelle salts, half an ounce each morning, in order to keep his bowels active, and pilocarpine, one-eighth grain hypodermatically, when necessary, to increase the action of the skin, in conjunction with hot-air baths. The last examination of the urine was made on the 21st of January, specific gravity then being 1008; number of ounces passed in twenty-four hours, sixty-two. The amount of albumen is unchanged. No casts observed under the microscope. The indications are that compensatory hypertrophy is being established, and our prognosis is that this man will improve for a time.

428 WASHINGTON BOULEVARD.

## Figures and Tables

**Figure f1:**